# The Enzymatic Antioxidants Activities Changes in Water Plants Tissues Exposed to Chlorpyrifos Stress

**DOI:** 10.3390/antiox11112104

**Published:** 2022-10-25

**Authors:** Elżbieta Sobiecka, Milena Mroczkowska, Tomasz P. Olejnik

**Affiliations:** 1 Institute of Natural Products and Cosmetics, Faculty of Biotechnology and Food Sciences, Lodz University of Technology, ul. Stefanowskiego 2/22, 90-357 Lodz, Poland; 2 Department of Sugar and Food Safety Management, Faculty of Biotechnology and Food Sciences, Lodz University of Technology, ul. Wolczanska 171/173, 90-530 Lodz, Poland

**Keywords:** chlorpyrifos, phytoremediation, macrophytes, enzymatic defense system

## Abstract

Water pollution is an immense environmental problem, and plant protection products are part of it. The organophosphorus insecticides, chlorpyrifos as an example, were used for years, and their high concentration could negatively influence ecosystems. Some of the plants, such as macrophytes, were exposed to a variety of stress factors. To live on, the macrophytes developed an efficient antioxidative system consisting of enzymatic and non-enzymatic antioxidants. The remediation process of polluted water ecosystems caused by plant protection products in our climate zone can be intensified if it is provided by autochthonic macrophytes. The results of our studies are part of the research that allows optimizing the phytoremediation process without irreversible effect on investigated species. The influence of various concentrations of chlorpyrifos on the enzymatic system in Canadian waterweed (*Elodea canadensis* Michx.), needle spikerush (*Eleocharis acicularis* L.), and water mint (*Mentha aquatica* L.) were studied. The differences in values of guaiacol peroxidase (GPX) and glutathione S-transferase (GST) activities were determined in leaves and roots. Research indicated an increase in both enzyme activities in plants exposed to toxic compounds. The highest concentration of chlorpyrifos affected the highest activities of enzymes. The water mint roots responded with the highest value of glutathione S-transferase activity during cultivation in polluted environment. It was therefore concluded that an aqueous plant exposed to the toxic insecticide created a defensive mechanism by enzymatic antioxidant systems that correlated to the pollutant concentration and plant species.

## 1. Introduction

While the production and utilization of plant protection products carry out benefits, they also have multiple adverse effects. The intensive use of pesticides in agriculture influences significantly the aquatic and terrestrial ecosystems, which may consequently result in changes in the organism’s living functions. The plant protection products or the compounds of their degradation are available in all types of flowing water—precipitation, surface water, and groundwater. The group that can affect the course of physiological processes of water plants is organophosphorus insecticides [1]. Chloropyrifos is one of the compounds that belongs to this group. In 1965, Dow Chemical Company started distributing this compound as a foliar pesticide [2].

The toxicity of organophosphate pesticides is based on inhibiting the activity of enzymes regulating the nervous system. They act as an inhibitor of the acetylcholinesterase enzyme decomposing acetylcholine and cholinesterase. These compounds permanently bind the hydroxylating group of the enzyme, which prevents the degradation of acetylcholine [3]. Blocking the activity of cholinesterase increases the level of acetylcholine and butyrylcholine. The state of hyperactivity and the paralysis of muscles and the central respiratory center appears [4]. Exposure to high concentrations of organophosphate pesticides may cause some symptoms such as nausea, headaches, dyspnea, uncoordinated movement, paralysis, and muscle spasms [5].

The presence of pesticides in the environment can affect the course of not only the physiological processes in plants, such as photosynthesis and respiration, but also the growth processes as well as cell division and synthesis of growth regulators. Moreover, damage to the chlorophyll synthesis pathway has been observed, and it showed that it leads to decreased content of this pigment in chloroplasts [6]. The occurrence of impurities may impair other physiological processes, e.g., water uptake or changes in the cell organelles structures, particularly in chloroplasts [7].

Water plants called macrophytes have developed an ability to accumulate pollutants inside their tissues. They are characterized by their growing and developing capabilities in difficult environmental conditions related to the presence of toxic substances. In order for them to be protected against oxidative stress, macrophytes have developed a system of antioxidant mechanisms consisting of nonenzymatic and enzymatic antioxidants.

The enzymatic antioxidant system is created by enzymes that carry out reactions to remove free radicals and prevent their formation; guaiacol peroxidase (GPX) and glutathione S-transferase (GST) are two of these enzymes [8,9].

The characteristic feature of peroxidases is their affinity for various types of xenobiotics, which enables them to participate in a number of detoxification processes [10]. They participate in various physiological processes, such as lignification, suberization, auxin catabolism, wound healing, and defense mechanisms against pathogen infections [11,12]. These enzymes may also take part in the immobilization of pollutants in the rhizosphere and in the oxidative degradation of compounds that are present in soil and water due to the ability of some plant species to secrete this enzyme into the root zone [13].

Guaiacol peroxidase is a glycoprotein, located in the cytosol, vacuole, cell wall, and intercellular space. It is associated with many important processes, such as lignification of the cell wall, degradation of indole-3-acetic acid, ethylene biosynthesis, wound healing, and defense against abiotic and biotic stress. The GPX is widely accepted as “stress enzymes”. It can act as an effective extinguisher for reactive oxygen intermediates and peroxide radicals under extreme conditions. It has been shown that different environmental stress conditions induce peroxidase activities in plant tissues [12,14].

Glutathione S-transferase is an enzyme catalyzing the coupling reaction of glutathione with nucleophilic and electrophilic compounds, resulting in conjugates with glutathione. The first report on the plant enzyme of glutathione S-transferase appeared in 1970. The GST catalyzes the detoxification of atrazine herbicide by its coupling with the endogenous glutathione tripeptide in sorghum and maize plants [15]. These preliminary results initiated intensive GST studies, which focused on the detoxification of various herbicides and other toxic xenobiotics compounds in plants [16,17].

The aim of our studies was to determine the activity changes of two antioxidant enzymes, guaiacol peroxidase (GPX) and glutathione S-transferase (GST), in the leaves and roots of selected macrophytes: Canadian waterweed (*Elodea canadensis* Michx.), needle spikerush (*Eleocharis acicularis* L.), and water mint (*Mentha aquatica* L.). The environmental stress tolerance was investigated in the cultures with various concentrations of chlorpyrifos and without an insecticide. The chosen plants are characteristic of the temperate zone in aquatic ecosystems. The results of our studies allow to optimize the phytoremediation process of the polluted aquatic environment using autochthonic macrophytes species.

## 2. Materials and Methods

### 2.1. Plants

Three macrophytes were investigated in our studies: the species of Canadian waterweed (*Elodea canadensis* Michx.), water mint (*Mentha aquatica* L.), and needle spikerush (*Eleocharis acicularis* L.) originated from organic farming.

All details concerning the cultivation process were described in the previously published paper of Sobiecka et al. [18]. The phytoremediation process was carried out in aquariums on aquarium gravel in an aqueous solution enriched with mineral salts: CaCl_2_ · 2H_2_O 0.106 g/dm^3^, MgSO_4_ · 7H_2_O 0.0038 g/dm^3^, Na_2_HPO_4_ · 12 H_2_O 0.0035 g/dm^3^, KH_2_PO_4_ 0.0138 g/dm^3^, Ca(HCO_3_)_2_ 0.272 g/dm^3^, FeSO_4_∙ 7H_2_O 0.0589 g/dm^3^, KCl 0.0038 g/dm^3^, and NaNO_3_ 0.022 g/dm^3^ in the temperature 23 ± 2 °C for 21 days in aerobic conditions. The experimental setup was provided in an aquarium with a day/night system at 21 ± 2 °C and a photoperiod of 14 h. The air humidity was measured by hydrometer (WSZ, Cracow, Poland). It was kept at 45–50% at a distance of 20 cm from the water surface.

The phytoremediation process was carried out in the aquatic environment polluted by different concentrations of chlorpyrifos: 50 μg/dm^3^, 100 μg/dm^3^, and 150 μg/dm^3^. Macrophytes were also cultivated in the medium without adding the tested insecticide as a reference test.

### 2.2. Determination of Glutathione S-transferase

The enzymatic antioxidants determination preceded the plant tissues homogenization in 0.05 mol/dm^3^ sodium phosphate buffer with pH 7.0. The MASTICATOR Basic lab blender (IUL, Barcelona, Spain) was used to prepare the homogenate. It was centrifuged for 15 min at 20,000 rpm in a centrifugal separator of Sigma type 2-16P (Polygen, PL). The supernatant was used to measure the enzymes activities.

Glutathione S-transferase activity was determined using Habig’s group modified method [19]. The mixture consisted of 0.1 mol/dm^3^ potassium-phosphate buffer (pH 6.25) and 30 mmol/dm^3^ GST reacted with 0.75 mmol/dm^3^, 1-chloro-2,4-dinitrobenzene (CDNB) to form a glutathione (GSH). The absorbance was measured at λ 340 nm using a UV–Vis spectrophotometer UV/VIS 8453 Spectroquant Nova 400 (Merck KGaA, Darmstadt, Germany) with optical glass vials (Supelco 1.14946, rectangular cells 10 mm) to observe the enzyme activity. The activity was calculated with the extinction coefficient of 9.6 mmol^−1^ cm^−1^ and expressed as nmol CDNB for 1 g of fresh mass (f.m.).

### 2.3. Determination of Guaiacol Peroxidase

The activity of the guaiacol peroxidase GPX was determined using the modified Maechly and Chance method [20]. The mixture consisted of 0.05 mol/dm^3^ acetate buffer (pH 5.6), 5 mmol/dm^3^ guaiacol, 15 mmol/dm^3^ H_2_O_2_, and 0.02 cm^3^ plant tissues extract, which reacted with enzymatic extract to form a tetraguaiacol (TG). The absorbance was measured at λ 470 nm using a UV–Vis spectrophotometer UV/VIS 8453 Spectroquant Nova 400 (Merck KGaA, Darmstadt, Germany) with optical glass vials (Supelco 1.14946, rectangular cells 10 mm) to observe the enzyme activity. The activity was calculated with the extinction coefficient of 26.6 mmol^−1^ cm^−1^ and expressed as mmol TG for 1 g of fresh mass (f.m.).

### 2.4. Statistical Analysis

The results obtained were analyzed statistically in STATISTICA Version 10 (StatSoft, Cracow, Poland). Three biological repetitions of measurements were averaged as the presented results. They were subjected to a single-factor analysis of ANOVA variance and then analyzed using Duncan multiple-range post hoc test (*p* < 0.05) in order to show statistically significant differences between the tested samples.

## 3. Results

**Figure 1:**
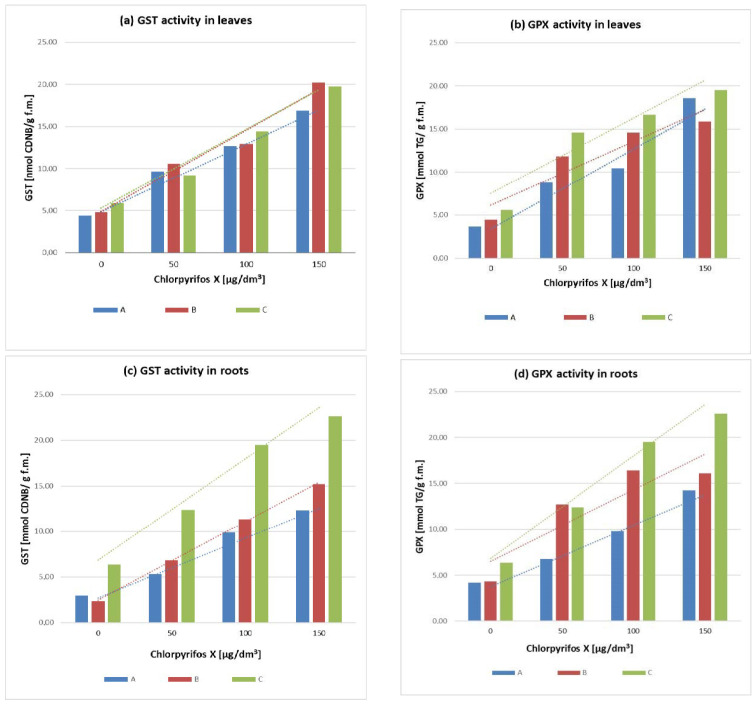
The enzyme activity changes: (**a**) GST activity in leaves; (**b**) GPX activity in leaves; (**c**) GST activity in roots; (**d**) GPX activity in roots in A—Canadian waterweed (*Elodea canadensis* Michx.), B—needle spikerush (*Eleocharis acicularis* L.), C—water mint (*Mentha aquatica* L.).

In the studies, the selected plants’ response to stress caused by various concentrations of chlorpyrifos was determined. The enzymatic oxidants’, glutathione S-transferase (GST) and guaiacol peroxidase (GPX), activities change in leaves and roots were analyzed. The results are presented in Figure 1.

Both of the enzyme activity changes in the roots and leaves of the tested plants were observed. Plants that were exposed to chlorpyrifos were characterized by higher GST and GPX activities compared to control cultivations of Canadian waterweed, needle spikerush, and water mint.

The highest activity of GPX was recorded for all plant species treated with the highest concentration of pesticide (150 µg/dm^3^). The increase was, respectively, more than 4-fold for the Canadian waterweed and more than 3-fold for the needle spikerush and water mint relative to plants grown under control conditions. In the case of the roots, the highest increase of GPX activity was observed for water mint growing in both cultures contaminated with the highest tested concentration of chlorpyrifos (150 µg/dm^3^). The growth was more than 5-fold for this plant compared to the control plants.

Increased GST activity changes were observed in the leaves and roots of all plants growing in the cultivated cultures in comparison to control plants. Analyzing the obtained results, a higher GST activity was recorded in leaves for plant species growing in cultures treated with the highest pesticide concentration (150 µg/dm^3^). In roots, the highest activity of this enzyme was indicated for water mint.

The effect of the chlorpyrifos contamination and its influence on the activities of glutathione S-transferase and guaiacol peroxidase in leaves and roots of the chosen macrophytes was investigated. The results allowed to calculate the model of the enzymes activities as the abiotic stress response. The model has been described with the equations presented in Table 1. The high value of the correlation coefficients R^2^ (Table 1) confirmed the proper use of the linear functions in the model description. Figure 1 includes the correlation curves as the graphical proof of the model calculations. The color of the curves corresponds to the colors of the columns in the individual diagrams for the plant species studied.

## 4. Discussion

The selected plants for the study—Canadian waterweed, needle spikerush, and water mint—showed an increase in antioxidant enzymatic systems activities as a response to abiotic stress caused by the presence of chlorpyrifos in the aquatic environment.

In cases of environmental stress, one of the first defensive reactions observed at the plants’ cells is the increase of reactive oxygen species concentration. The plants’ response is a development of an efficient antioxidative system consisting of enzymatic and nonenzymatic antioxidants [21]. The research studies on abiotic stress caused by the presence of toxic organic compounds related to the increased activity of enzymatic antioxidants [22,23]. The enzymatic oxidants system consisted of few enzymes, such as superoxide dismutase, ascorbate peroxidase, catalase, glutathione reductase, as well as guaiacol peroxidase and glutathione S-transferase [24]. The abiotic stress caused by the presence of chlorpyrifos altered the ascorbate-glutathione cycle (Asc-Glu), causing changes in the cellular balance of ion exchange [25].

The research of Parween et al. [26] referred to the increase of enzymes activities during 15 days of *Vigna radiata* L. cultivation in the aquatic environment polluted by chlorpyrifos. At the end of the experiments, the activities all the investigated enzymes decreased. We observed that in the 21st day of cultivation, the activities of guaiacol peroxidase and glutathione S-transferase still showed growth. It could be explained by the experimental setup, such as the composition of aquatic medium. In our research, the mineral ions added to the medium delayed the negative effect of chlorpyrifos to plants.

Glutathione S-transferase (GST) is responsible for the detoxification of reactive products formed during oxidative stress and participates in the process of detoxification of xenobiotics such as carcinogens, heavy metals, or pesticides. The plant defends itself against the accumulation of pollutants in the cytoplasm and transports the xenobiotics conjugates by means of tonoplast collecting them inside the vacuole. Then, they are deposited in the cell wall, where they merge with lignins and hemicelluloses and are eventually secreted outside the cell (e.g., into the rhizosphere) [27].

Similar results for GST activities were obtained in studies conducted by Tlidjen [28] on Canadian water weed and common duckweed growing in an environment contaminated with herbicide of different concentrations, whose active substance was methyl diclofop (35 µg/dm^3^, 70 µg/dm^3^, and 140 µg/dm^3^). Herbicide was added on the 7th and the 21st day of cultivation. As a result of exposure to herbicide, a significant increase in glutathione S-transferase activity was observed in the common duckweed on day 21 treated with the highest concentration of methyl diclofop. In the case of Canadian waterweed, the highest increase in glutathione S-transferase activity was observed on the 7th and 21st day of culture exposed also to the highest herbicide concentration.

The obtained results suggest that the highest GST activity was observed in the roots, which are most exposed to pesticides compared to other plant organs. A clear increase in the activity of glutathione S-transferase was recorded in the roots of plants, which may indicate the interaction of macrophytes with microorganisms in the process of removing pesticides from the environment. This activity may be related to the contribution of the enzyme to the removal of toxic aldehydes formed as degradation products of lipid peroxides. These changes indicate the occurrence of oxidative stress due to the accumulation of reactive oxygen species inducing the lipid peroxidation process, leading to structural damage and loss of cell membrane integrity [29].

The second of the determined enzymes—guaiacol peroxidase—belongs to the enzymes that are responsible for the proper course of physiological processes in plant tissues. Peroxidases are characterized by inactivating reactive oxygen species. Their action involves catalyzing the decomposition reaction of toxic substances. Among all peroxidases, changes in guaiacol peroxidase activity caused by oxidative stress did not dominate in the scientific literature. Nevertheless, this enzyme was recognized by Noctor and Foyer as one of the most important antioxidant enzymes in plant cells [30].

Similar results of GPX activities were obtained in studies conducted by Bertrand [31], in which different concentrations of chlorpyrifos were tested (3.5 ng/L; 10.5 ng/L; 31.5 ng/L and 94.5 ng/L) in culture in small pondweed (*Potamogeton pusillus*) tissue. The degree of POX activity was dependent on the pesticide concentration. The authors noted an increase in the activity of guaiacol peroxidase both in leaves and roots of the plant exposed to the highest concentration of chlorpyrifos (94.5 ng/L), with the highest POX content observed in the roots that were most exposed to the pesticide. The results obtained show that guaiacol peroxidase is an antioxidant enzyme, and its increased activity is correlated with the occurrence of oxidative stress in plant tissues.

Synchronous increase in the activity of guaiacol peroxidase and glutathione S-transferase in plant tissues growing under the conditions of exposure to chlorpyrifos may indicate the interaction of these enzymes during prevention and removal of oxidative stress effects. The GPX and GST are involved in the process of detoxification of cells from organic peroxides and hydrogen peroxide, which are products of aerobic metabolism [32]. Moreover, some plants secrete significant amounts of peroxidases to the rhizosphere, where they participate in oxidative degradation of toxic compounds and in reactions leading to immobilization of pollutants in the rhizosphere [33].

## 5. Conclusions

The described studies proved that investigated macrophytes, i.e., Canadian waterweed (*Elodea canadensis* Michx.), water mint (*Mentha aquatica* L.), and needle spikerush (*Eleocharis acicularis* L.), exposed to toxic insecticide molecules created a defensive mechanism by enzymatic antioxidant systems. The enzymes activities depended on the pollutant concentration and water plant species as well as the type of plant’s tissue.

The results allowed to observe the linear correlation between the number of toxic compounds and the investigated enzymes activities. The increased concentrations of chlorpyrifos resulted in an increase in the activities of glutathione S-transferase and guaiacol peroxidase, which perform detoxification functions in the roots of the tested plants. Research indicates that the environment contaminated with chlorpyrifos affects the level of antioxidants and dyes metabolized by plants. Increasing concentrations of chlorpyrifos resulted in boosting the activity of enzymes such as GST and GPX, which have detoxification functions in the roots of the studied plants.

## Figures and Tables

**Table 1:** The equations and correlation coefficients for the model describing changes in enzymes activities.

Enzymatic Antioxidant	Plant	Equation	R^2^
GST in leaves	A	f_A_(x) = 4.048 x + 0.790	0.9894
	B	f_B_ (x) = 4.858 x + 0.005	0.9660
	C	f_C_(x) = 4.689 x + 0.605	0.9897
GST in roots	A	f_A_(x) = 3.281 x − 0.595	0.9828
	B	f_B_(x) = 4.319 x − 1.865	0.9988
	C	f_C_(x) = 5.588 x + 1.245	0.9784
GPX in leaves	A	f_A_(x) = 4.639 x + 1.225	0.9378
	B	f_B_(x) = 3.708 x + 2.430	0.8745
	C	f_C_(x) = 4.378 x + 3.140	0.8856
GPX in roots	A	f_A_(x) = 3.332 x + 0.435	0.9842
	B	f_B_(x) = 3.899 x + 2.640	0.8016
	C	f_C_(x) = 5.588 x + 1.245	0.9784

A—Canadian waterweed (*Elodea canadensis* Michx.), B—needle spikerush (*Eleocharis acicularis*), C—water mint (*Mentha aquatica* L.).

## Data Availability

The data are contained within the article.
